# New Insights into Signed Path Coefficient Granger Causality Analysis

**DOI:** 10.3389/fninf.2016.00047

**Published:** 2016-10-27

**Authors:** Jian Zhang, Chong Li, Tianzi Jiang

**Affiliations:** ^1^School of Mathematical Sciences, Zhejiang UniversityHangzhou, China; ^2^Brainnetome Center, Institute of Automation, Chinese Academy of SciencesBeijing, China

**Keywords:** signed path coefficient, Granger causality, fMRI, model order, vector autoregression

## Abstract

Granger causality analysis, as a time series analysis technique derived from econometrics, has been applied in an ever-increasing number of publications in the field of neuroscience, including fMRI, EEG/MEG, and fNIRS. The present study mainly focuses on the validity of “signed path coefficient Granger causality,” a Granger-causality-derived analysis method that has been adopted by many fMRI researches in the last few years. This method generally estimates the causality effect among the time series by an order-1 autoregression, and defines a positive or negative coefficient as an “excitatory” or “inhibitory” influence. In the current work we conducted a series of computations from resting-state fMRI data and simulation experiments to illustrate the signed path coefficient method was flawed and untenable, due to the fact that the autoregressive coefficients were not always consistent with the real causal relationships and this would inevitablely lead to erroneous conclusions. Overall our findings suggested that the applicability of this kind of causality analysis was rather limited, hence researchers should be more cautious in applying the signed path coefficient Granger causality to fMRI data to avoid misinterpretation.

## 1. Introduction

Granger causality analysis is an important time series analysis technique that originally derived from econometrics. In recent years it has been widely employed across the field of neuroscience, especially for constructing effective networks among brain regions in fMRI causal modeling studies (Friston, 2009, 2011; Bressler and Seth, 2011; Valdes-Sosa et al., 2011; Friston et al., 2012; Stephan and Roebroeck, 2012). The basic idea of Granger causality can be traced to Norbert Wiener (1956) and a practical implementation was proposed by Clive Granger (1969) in the framework of linear autoregressive model. The concept is based on the idea that the “cause” will precede and help to improve the prediction of the “effect.” As a broadly applied time series analysis method for assessing directional influence in neuroscience data, Granger causality has been proven to be useful and informative, while on the other hand it has also drawn a lot of debates about its applicability and interpretation in fMRI studies. Previous discussions around the controversial aspects of application of Granger causality to fMRI data are mostly focused on hemodynamic response latency, low sampling rate or measurement noise (David et al., 2008; Witt and Meyerand, 2009; Smith et al., 2011; Webb et al., 2013; Friston et al., 2014). Various factors could impact the detectability of the neuronal interactions by Granger causality analysis in fMRI, and a great deal of detailed literature has been written on this topic. Some simulation studies pay attention to hemodynamic response variability on Granger causality at the level of group (Schippers et al., 2011; Smith et al., 2012), others focus on the effect of signal-to-noise ratio or the sampling period (Deshpande et al., 2010), also there is investigation addressing this problem by a rigorous combination of theory and simulation modeling at multiple levels of biophysical details, showing that severe downsampling may render Granger causality to follow the hemodynamics rather than the neural mechanisms (Seth et al., 2013), and to cope with the hemodynamic response variability some researchers propose to use the difference of Granger causality influence term rather than each direction separately (Roebroeck et al., 2005). Although these discussion is far from settled, and miscellaneous factors may corrupt Granger causality which lead to confounding results, highly interpretable applications of Granger causality to fMRI continue to appear, hence it is still widely considered as a viable technique for analyzing fMRI data (Wen et al., 2013).

In the present study, we were mainly concerned with an alternative formulation of Granger causality, the “signed path coefficient” method. In the most commonly used formulations of Granger causality (Ding et al., 2006), the degree to which a causal variable help to predict an effective variable beyond the information contained in the effective variable's own past is measured either by the decrease of the residuals (Ding et al., 2006; Jiao et al., 2014; Kullmann et al., 2014), i.e., the time domain formulation, or by the frequency decomposition (Geweke, 1982; Bajaj et al., 2014, 2015), i.e., the frequency domain formulation, both through estimating an autoregression model with a fixed order. However, in some recent studies, another “signed path coefficient” version of Granger causality was proposed (Chen et al., 2009) and had been firstly used to reveal the causality from brain regions of patients with major depression disorder (Hamilton et al., 2011), and gradually it was popular among a number of researchers in the field of fMRI. Some researchers had integrated this idea in their toolkit (Zang et al., 2012) and a great deal of papers had applied it to analyze effective networks in fMRI data (Ji et al., 2013; Palaniyappan et al., 2013; Abe et al., 2015; Wu et al., 2015; Zhang et al., 2015; Feng et al., 2016; Yuan et al., 2016). Generally speaking, this coefficient-based causality method characterizes the strength and direction of causal influence among ROIs or voxels in fMRI data by directly computing the signed path coefficients through an order-1 autoregression model, and in this process the positive or negative coefficients are respectively defined as excitative or inhibitive influences that one brain region cast to another. Therefore, many authors in recent years prefer this method for two reasons: first, it is easy to implement with a relatively low computational burden because it only requires to estimate the lag-1 coefficient matrix; second and the most important, compared with the traditional (with on sign) Granger causality that based on residuals, this new method could distinguish an excitatory/inhibitory relationship among brain regions (Hamilton et al., 2011) by means of a positive/negative sign of coefficient, which provides a convenient neural interpretation for the final conclusion. Different from the classic Granger causality method which aims to measure the “causal effect” between time series, the signed path coefficient method is actually a measure of “causal mechanism” (Barrett and Barnett, 2013), and in this paper we will point out there exists some ambiguous aspects about the excitatory/inhibitory definition in this kind of denationalization of Granger causality which needs to be clarified.

The present study's aim is to investigate under what condition the signed path coefficient Granger causality will meet some bias or induce false interpretation as a method in causality inference. To do this, we test the signed path coefficient approach in fMRI data and perform a series of simulations to elucidate the unproper application of this method.

## 2. Granger causality

The original Granger causality formalization introduced by Granger (1969) is based on the linear autoregression framework, in which the degree of predictors minimizing the forecast mean squared errors (MSEs) is measured as the causality strength. To illustrate this in bivariate context, consider two jointly stationary stochastic processes *X*_*t*_ and *Y*_*t*_, which could be individually described by an autoregression representation (Ding et al., 2006).

(1) $$\begin{array}{l} X_{t}=\sum_{j\;=\;1}^{p}a_{0j}X_{t\;-\;j}+\epsilon_{0t}\\ \;Y_{t}=\sum_{j\;=\;1}^{p}d_{0j}Y_{t\;-\;j}+\eta_{0t} \end{array}$$

One the other hand, the two process *X*_*t*_ and *Y*_*t*_ could be jointly represented as:

(2) $$\begin{array}{l} X_{t}=\sum_{j\;=\;1}^{p}a_{1j}X_{t\;-\;j}+b_{1j}Y_{t\;-\;j}+\epsilon_{1t}\\ Y_{t}=\sum_{j\;=\;1}^{p}c_{1j}X_{t\;-\;j}+d_{1j}Y_{t\;-\;j}+\eta_{1t} \end{array}$$

The time lags parameter *p* is the model order that can be determined by some information criteria to reach a parsimonious model through the trade-off between bias and variance (Burnham and Anderson, 2002). To apply in computation we need to pick a finite order *p*, which is equivalent to place zero-restrictions uniformly on the rest of coefficients with the lags larger than *p* (Lütkepohl, 2005). The traditional Granger causality measure is define as the ratio between the variances of the residual errors, which could be easily extended to the multivariate process.

(3) $$\begin{array}{l} F_{X\to Y}=log\left(\frac{var(\eta_{0t})}{var(\eta_{1t})}\right)\\ F_{Y\to X}=log\left(\frac{var(\epsilon_{0t})}{var(\epsilon_{1t})}\right) \end{array}$$

In the method of signed path coefficient Granger causality which is introduced by Chen et al. (2009) and has been employed in a series of recent fMRI studies (Hamilton et al., 2011; Zang et al., 2012; Ji et al., 2013; Palaniyappan et al., 2013; Kullmann et al., 2014; Abe et al., 2015; Wu et al., 2015; Zhang et al., 2015; Feng et al., 2016; Yuan et al., 2016). The causality strength between time series is measured not by the change of the residuals, but directly by the regression coefficients in (2.2) through an order-1 autoregression model (Hamilton et al., 2011), without considering the higher lags (i.e., *p* = 1). In this case, the time series *X*_*t*_ significantly Granger causes the time series *Y*_*t*_ if the signed path coefficient *c*_11_ is significantly larger or smaller than zero, and vice versa, the causal influence from *Y*_*t*_ to *X*_*t*_ is measured by the coefficient *b*_11_. According to this formulation, any significant positive/negative coefficient is interpreted as an excitatory/inhibitory interaction among brain regions (Hamilton et al., 2011). An advantage of the signed path coefficient method is the direct computation of an autoregressive model, without resorting to the restricted model (Seth, 2010). That means the terms in (2.1) are irrelevant and can be neglected.

Nevertheless, in this kind of causality analysis procedure, an essential factor which is critical to the interpretation of the final result has been usually neglected by the researchers: will the optimal model order which is used to construct the autoregressive model (under bivariate or multivariate condition) always be one? And if not, how the outcome will be impacted? In the present work our primary purpose is to gain an insight into the validity of this form of causality analysis. And we will show that the signed path coefficients are not always consistent with the underlying causal relationships if the actual order of the real data generation process is larger than one but the analysis is processed by estimating an underfitted order-1 autoregressive model. Here we are trying to clarify two points: first, the procedure to determine the optimal order of the time series to be analyzed is imperative, inasmuch as the signed path coefficient method focuses on the correct estimation of parameters, so it must be verified whether the choice of order-1 autoregressive model is sufficient to reconstruct the underlying causality relationships; second, if we need to consider more information of higher lags to make correct inference from the data, does the assumption that positive/negative coefficients indicating the excitatory/inhibitory interaction still holds? Actually from some simulation results we will show that, under certain situations the signs of the path coefficients are not consistent with the real causal relationships, which means for higher order autoregressive models the interpretation that positive/negative coefficients as excitatory/inhibitory influence could hardly hold.

In this paper our statistical analysis for the coefficient Granger causality was performed by non-parametric methods, which involved permutation resampling of the original data for constructing surrogates to assess whether a path coefficient value was significantly different from zero (i.e., a two-tailed permutation test with a *p*-value of at least 0.05). In the resampled data the the order of the time points had been shuffled to destroy any underlying causality structure. And significance thresholds were derived by examining the empirical quantiles of the distribution of regressive coefficients.

## 3. Materials and methods

### 3.1. Resting-state fMRI data

To illustrate the problem of signed path coefficient Granger causality, we started with an identification of the optimal order in fMRI data with different TR times. The following causality analysis were performed on a publicly released resting-state fMRI dataset: the enhanced Nathan Kline Institute-Rockland sample from “1000 Functional Connectomes Project” http://fcon_1000.projects.nitrc.org/indi/pro/eNKI_RS_TRT. Two resting-state fMRI data sets with different TR (*TR* = 0.645 s, 3 mm isotropic voxels, 10 min; and *TR* = 1.4 s, 2 mm isotropic voxels, 10 min) were acquired on Siemens 3T Trio Tim scanners. All participants had no history of neurological and psychiatric disorders and all gave the informed consent approved by local Institutional Review Board.

### 3.2. Data preprocessing

Preprocessing of the resting-state fMRI data was performed using Statistical Parametric Mapping 8 (SPM8, http://www.fil.ion.ucl.ac.uk/spm) and Data Processing Assistant for Resting-State fMRI (DPARSFA, http://rfmri.org/DPARSF; by Yan and Zang, 2010). The first ten volumes of functional images were discarded to allow stability of the longitudinal magnetization, then the data were realigning with the corresponding T1-volume, spatial normalization into the stereotactic space of the Montreal Neurological Institute and resampling to 3-mm isotropic voxels, linearly detrended, followed by nuisance covariate regression (six head motion parameters, global mean signal, white matter signal and cerebrospinal fluid signal). Three subjects were excluded from further analysis because of head movement exceeding 1.5 mm or 1.5 degree in rotation.

With respect to bandpass filtering, it has been demonstrated (Barnett and Seth, 2011) this procedure has critical impact on the Granger causality analysis, disturbs the causal information and leads to spurious or missed causality. This kind of effect has been thoroughly discussed by former researchers (Florin et al., 2010; Barnett and Seth, 2011), however some fMRI study that adopt signed path coefficient causality method (Ji et al., 2013; Palaniyappan et al., 2013; Abe et al., 2015; Wu et al., 2015; Zhang et al., 2015; Feng et al., 2016; Yuan et al., 2016) still apply this procedure during preprocessing while a few others not (Hamilton et al., 2011; Wu et al., 2011). To investigate the influence of bandpass filtering to the signed path coefficient causality we will compare the both results of the preprocessed data with and without bandpass filtering procedure.

Finally, the functional images were segmented into 90 regions of interest (ROI) using automated anatomical labeling (AAL) template and the respective time series of each ROI was obtained by averaging the fMRI signals across all voxels in the ROI. Spatial smoothing was not performed for it might blur the boundary among the brain regions, but it is not crucial to the conclusion of this study, actually the following analysis will not meet any difference in the smoothed data and we just do not include that.

### 3.3. Model order determination

The construction of the autoregressive model requires to determine the order *p* to make sure a satisfactory amount of coefficients to fit the data optimally. There are many propositions and discussions in econometrics field around this topic, and the most used two kinds of information criteria are: Akaike Information Criteria (AIC) which is based on the Kullback-Leibler information, aims to make the best forecast precision; and Bayesian Information Criteria (BIC) which is based on the dimension-consistency, aims to select the true model order. The former is more biologically rational compare with the later since the brain is such a complex system and no “true model” could be expected to exist. And moreover, the usual limited length of fMRI data is not large enough for the dimension-consistent criteria to converge (Umbach and Wilcox, 1996; Burnham and Anderson, 2002). For this reason in fMRI analysis AIC shall perform better than BIC, whose penalty term is more strict and tend to give an underfitted model.

The formulation of small-sample bias adjustment AIC: AICc (Hurvich and Tsai, 1989) which is used in the following computation is:

(4) $$AICc=T*\ln(det(\Sigma ))+2K*\frac{T}{T-K-1}$$

and the formulation of BIC is:

(5) BIC=T∗ln(det(Σ))+K∗log(T)

where *T* is the data length, Σ is the maximum likelihood estimator obtained by fitting an autoregressive model (Lütkepohl, 2005), and *K* = p^*^n^2^ is the total number of estimated regression parameters, which increases in the penalty term by *n*^2^ in both criteria, i.e., the more time series of ROIs are considered, the more likely a small number will be chosen as the optimal order by AIC/BIC.

## 4. Results

### 4.1. Results of order-1 autoregression to fMRI data

**Figure 1:**
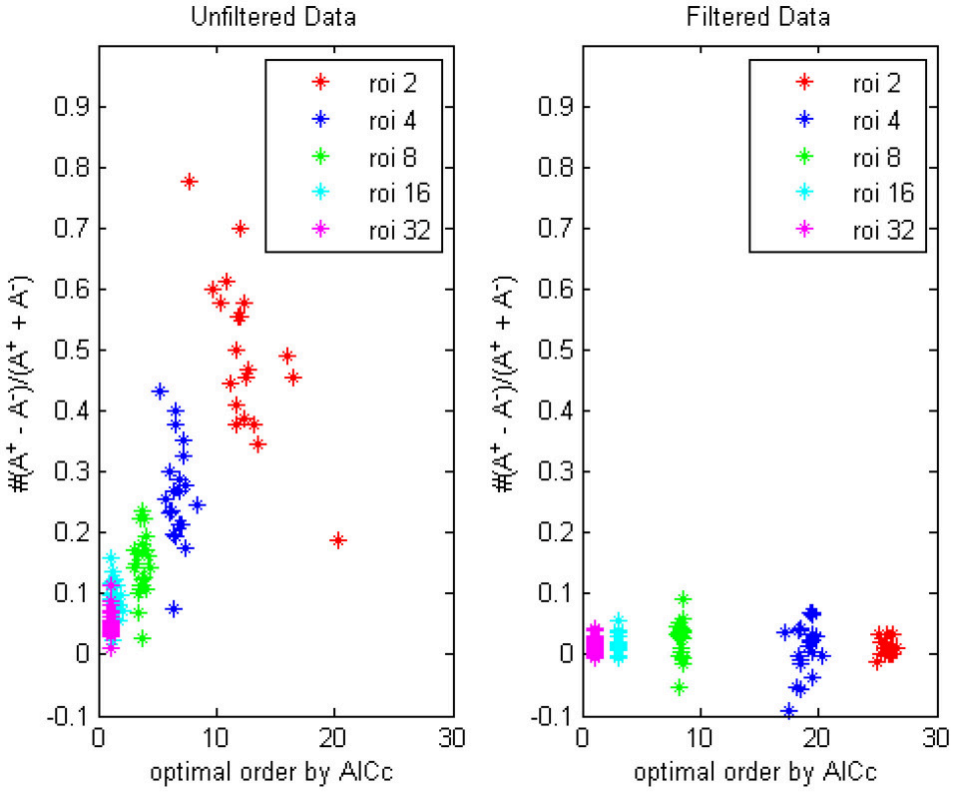
**The trending of the optimal model order by AICc and the coefficients bias for different ROI combinations, on filtered and unfiltered fMRI data**. *TR* = 645 ms.

First by AICc criteria we computed the optimal model orders and (non-diagnal) order-1 autoregressive coefficients of respectively 2, 4, 8, 16, and 32 ROIs, each group for 90 combinations (for example, AAL k to AAL *k* + 31 (mod90), for *k* = 1 to 90) and the value were averaged for every person, on filtered and unfiltered data, the result was shown in Figure 1 (*TR* = 0.645 s). The difference of the numbers of positive coefficients and negative coefficients (*A*^+^−*A*^−^)/(*A*^+^+*A*^−^) indicated that the fewer time series were considered, the bias of coefficients toward positive value would become more prominent, which implied more “excitatory” influences existing, in the following section we would try to give an explanation of this phenomenon through some simulations.

**Figure 2:**
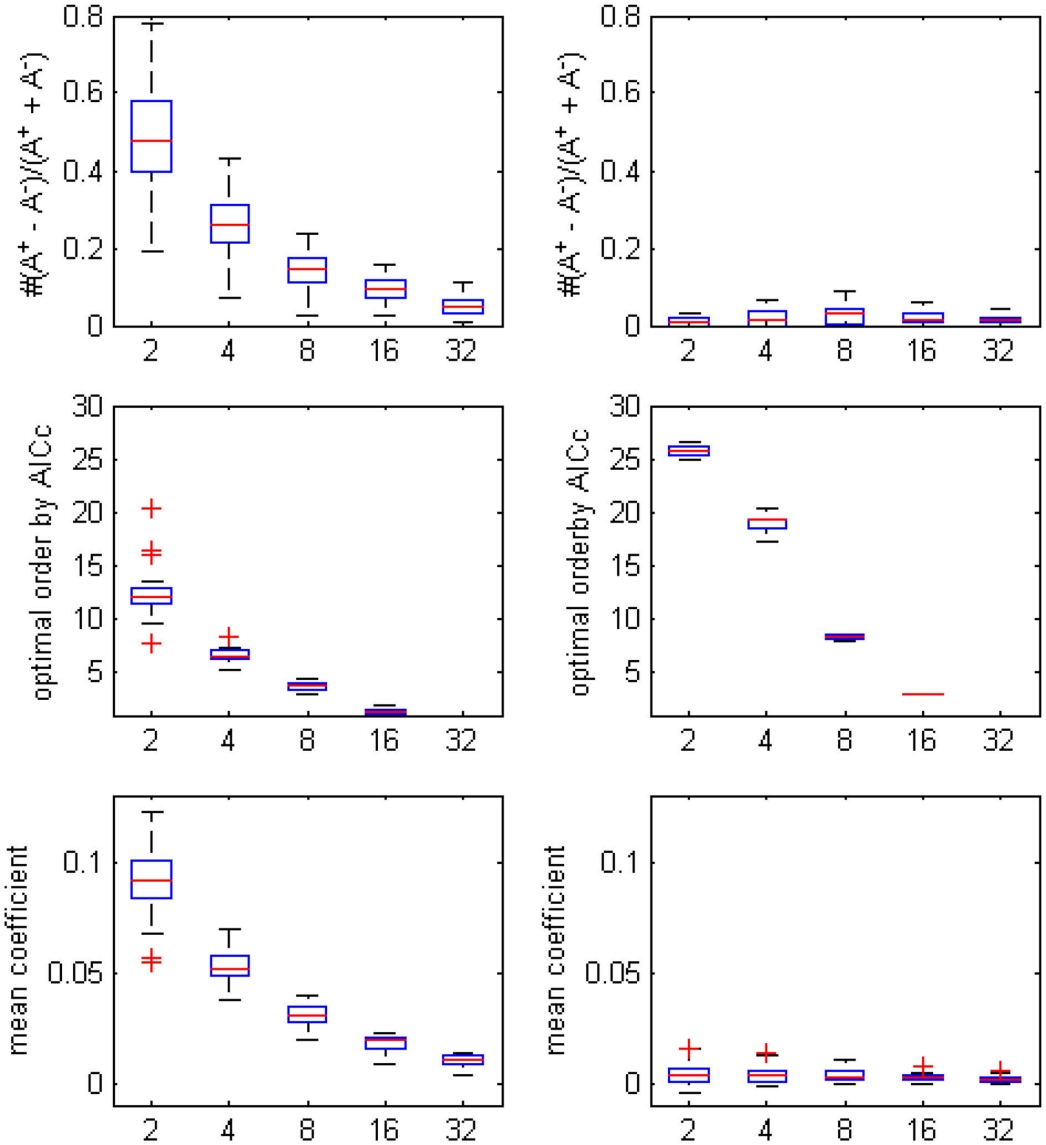
**The boxplot of the optimal model order by AICc, the coefficients bias and the mean coefficients for different ROI combinations, on filtered and unfiltered fMRI data**. *TR* = 645 ms.

The optimal model order that was determined by AICc turned to be lower when more ROI time series were considered in the autoregression, since the penalty term in the criteria formula increased rapidly with the square of number of time series and the likelihood of choosing a small *p* became dominant (the boxplot in Figure 2. showed the decrease trending of the orders and coefficients respectively). Compared with the unfiltered data, the model orders computed from filtered data were larger, which was in accord with the conclusion of Barnett and Seth (2011) that the filtering procedure will induce an increase in the model order, leads to model mis-specification under very limited data and may corrupt the empirical estimates. On the other side, the bias of positive coefficients disappeared, thus consolidated the causality structure has been distorted after the filtering.

**Figure 3:**
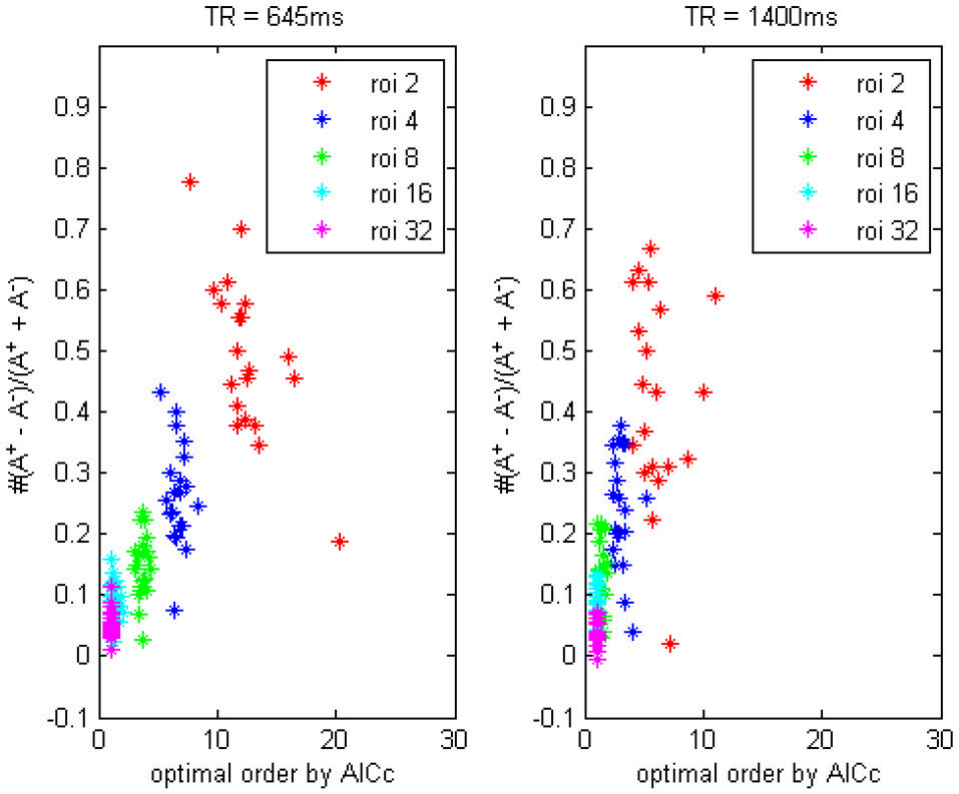
**The trending of the optimal model order by AICc and the coefficients bias for different ROI combinations, on fMRI data of *TR* = 645 ms and *TR* = 1400 ms**.

In Figure 3 we compared the difference between data with *TR* = 0.645 and 1.4 s, it showed the TR time could also affect model order determination. Shorter TR always induces a higher order and vice versa, thus to reach an optimally fitted model it is rationally to use different order for data under different TR set, this important factor has been generally overlooked by a remarkable number of researchers, who always simply choose to use the order-1 model across miscellaneous fMRI data, which will inevitably lead to some kind of erroneous conclusions. To address this issue we would provide some simulation examples in the next section to illustrate the application of signed path coefficient method will indeed lead to mistaken identification of causality.

**Figure 4:**
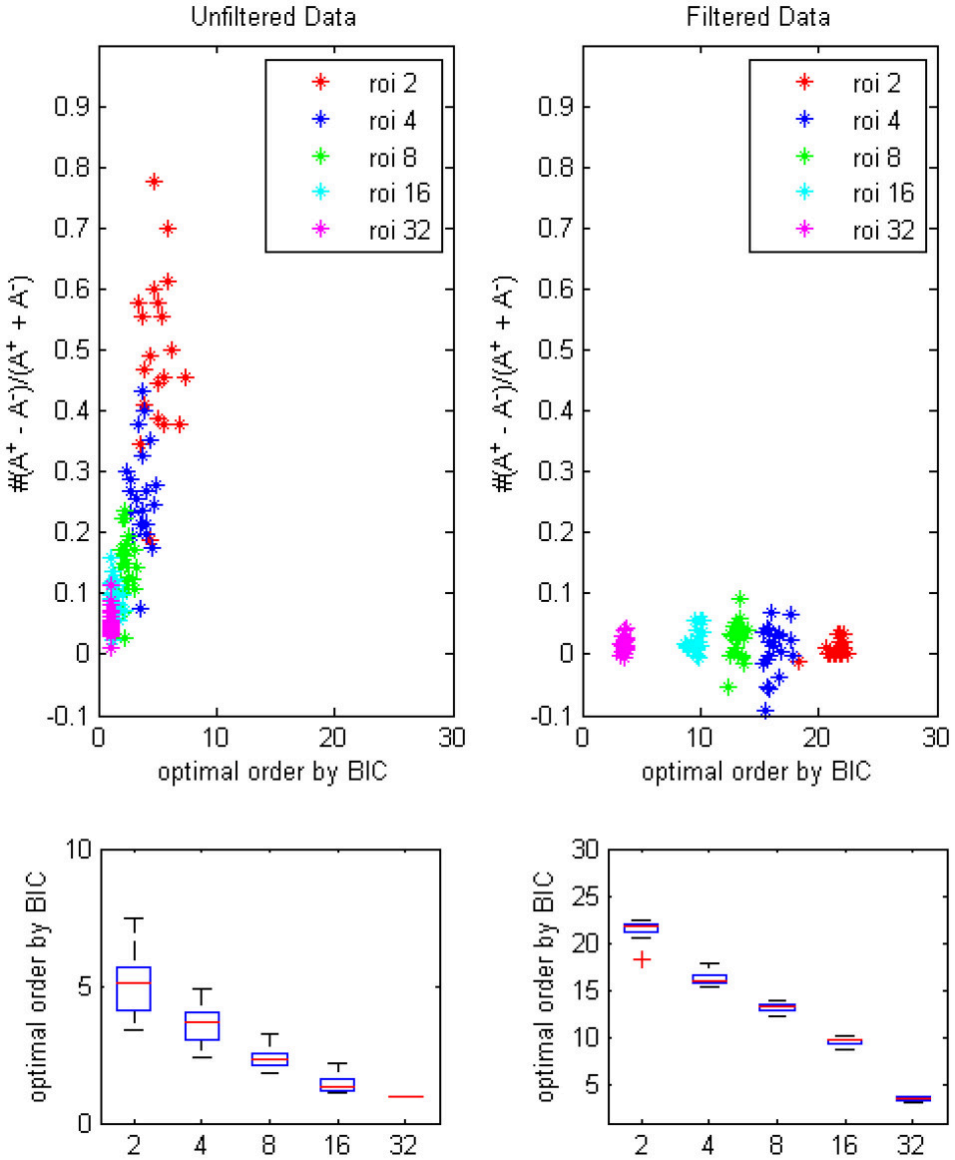
**The trending of the optimal model order by BIC and the coefficients bias for different ROI combinations, on filtered and unfiltered fMRI data**. *TR* = 645 ms.

The results of BIC (Figure 4) was similar to the result of AICc except that optimal orders of different ROI combinations were lower due to the more strict penalty term in the formulation of BIC.

### 4.2. Results of simulations

In this section we construct some bivariate time series models of which the underlying data generating processes are all autoregressive models with an optimal order higher than one, to manifest that in certain cases the choice of order-1 autoregression to fit the data is improper and may cause erroneous results, since the signed path coefficient Granger causality will be contradictory to the real causal relationship between the time series. Briefly speaking, the definition of excitatory/inhibitory influences to the positive/negative coefficients shall mostly be restricted to order-1 autoregression coefficient method in the case of an order-1 data generating process, and when we use an order-1 (underfitted) model to the data generated by a higher order process, the coefficient method is often not able to correctly identify the underlying causal structure, this fact illustrates why we should be rather cautious to the application of this kind of causality analysis.

Model 1. Here we start with an order-3 bivariate autoregression process with unit variance Gaussian noise. The model is:

(6) $$\begin{array}{l} \left(\begin{array}{c} X_{t}\\ Y_{t} \end{array}\right)=\left(\begin{array}{cc} 0 & 0\\ 0 & 0 \end{array}\right)\left(\begin{array}{c} X_{t-1}\\ Y_{t-1} \end{array}\right)+\left(\begin{array}{cc} 0 & 0.6\\ -0.6 & 0 \end{array}\right)\left(\begin{array}{c} X_{t-2}\\ Y_{t-2} \end{array}\right)\\ \;+\;\left(\begin{array}{cc} 0 & c\\ 0 & 0 \end{array}\right)\left(\begin{array}{c} X_{t-3}\\ Y_{t-3} \end{array}\right)+\left(\begin{array}{c} \epsilon_{x,\;t}\\ \epsilon_{y,\;t} \end{array}\right) \end{array}$$

**Figure 5:**
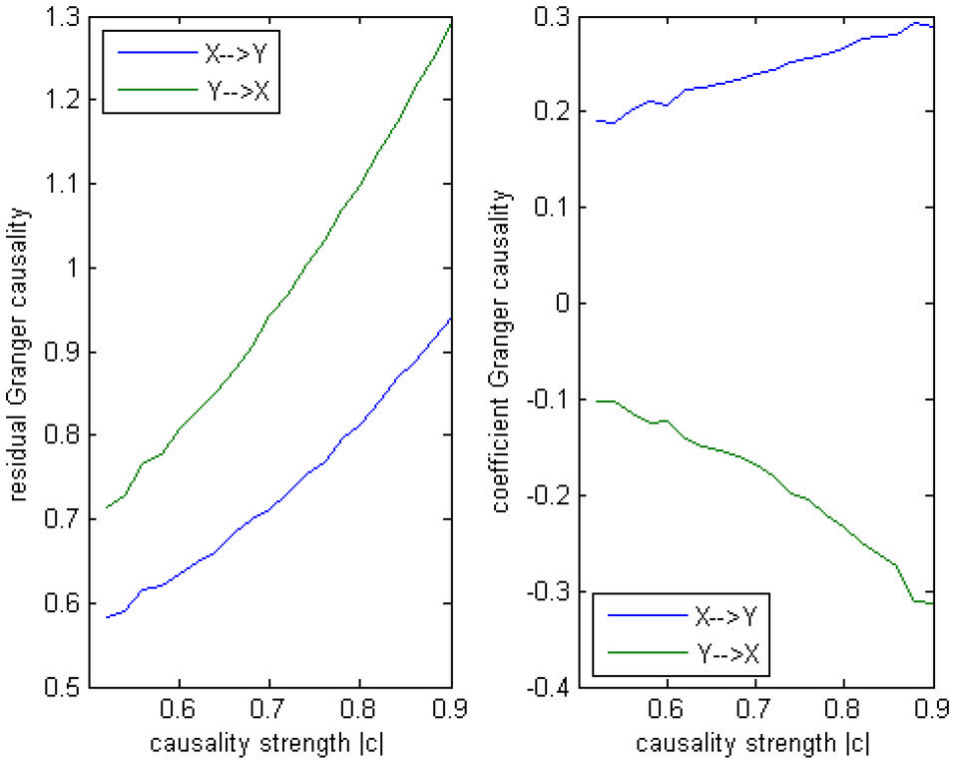
**Granger causality results from Model 1**. **Left:** result from traditional residual causality under optimal order. **Right:** result from order-1 signed path coefficient causality.

**Figure 6:**
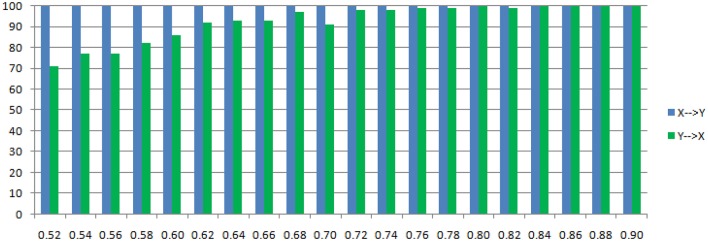
**Statistically significant percentage of connections between X and Y under permutation test for Model 1**. For each strength of c (x-axis), the number of significant results were showed by 100 simulations.

Where c is a negative influence on the order-3 from *Y* to *X* whose strength varies from −0.5 to −0.9 with a step size of 0.02 (*c* = −0.5−0.02^*^k), so that both AICc and BIC will give an optimal order no less than 3 (Table 1, averaged from 100 simulations with data length 1000). On order-1 there is not any interaction between *X* and *Y*, and on order-2 two causal influences exist with the same strength and opposite signs (+0.6/−0.6). However, it is impossible to deduce an excitatory/inhibitory influence from this model by the coefficient causality method, because the autoregression coefficients produce a completely contrary result. From outcome of 100 simulations it can be noticed that the signed path coefficient causality (Figure 5, right part) of *X* to *Y* was positive and increased with the absolute value of c, while the actual order-2 influence of X to Y was an opposite −0.6, hence in this example the coefficient method could not correctly detect the interactions on the order that higher than one. The problem was derived from the underfitted order-1 model being applied to the higher data generation process. On the other side, the coefficient of *Y* to *X* was negative and decreased with the absolute value of c, while the actual order-2 influence of *X* to *Y* was 0.6, jointly with an negative order-3 influence c, which apparently could not simply be described as an inhibitory interaction since that will undoubtedly made this kind of relationship confused. As the strength of c increased the coefficients became larger that rendered this misleading effect more significant (Figure 6), and we can see the different performance between the residual Granger causality (Figure 5, left part) and coefficient Granger causality. To make this point more clear, let us construct the following order-1 model (Model 1b) in which the coefficients are generated in a way to approximate the outcome derived from Model 1 by coefficient method:

**Table 1:** **Granger causality results from Model 1**.

**C**	**#A+**	**#A−**	**AICc**	**BIC**
−0.52	101	99	3.14	3
−0.54	100	100	3.21	3
−0.56	100	100	3.14	3
−0.58	100	100	3.31	3
−0.60	101	99	3.2	3
−0.62	100	100	3.21	3
−0.64	100	100	3.22	3
−0.66	100	100	3.28	3
−0.68	100	100	3.25	3
−0.70	100	100	3.26	3
−0.72	100	100	3.16	3
−0.74	100	100	3.24	3
−0.76	100	100	3.36	3
−0.78	100	100	3.34	3
−0.80	100	100	3.29	3
−0.82	100	100	3.34	3
−0.84	100	100	3.36	3
−0.86	100	100	3.26	3
−0.88	100	100	3.2	3
−0.90	100	100	3.25	3

Number of positive/negative coefficients and the average AICc/BIC in 100 simulations under different causality strength c.

Model 1b.

(7) $$\begin{array}{l} \left(\begin{array}{c} X_{t}\\ Y_{t} \end{array}\right)=\left(\begin{array}{cc} 0.5 & -0.071-0.011*k\\ 0.186+0.005*k & 0.5 \end{array}\right)\left(\begin{array}{c} X_{t\;-\;1}\\ Y_{t\;-\;1} \end{array}\right)\\ \;+\;\left(\begin{array}{c} \epsilon_{x,\;t}\\ \epsilon_{y,\;t} \end{array}\right) \end{array}$$

**Figure 7:**
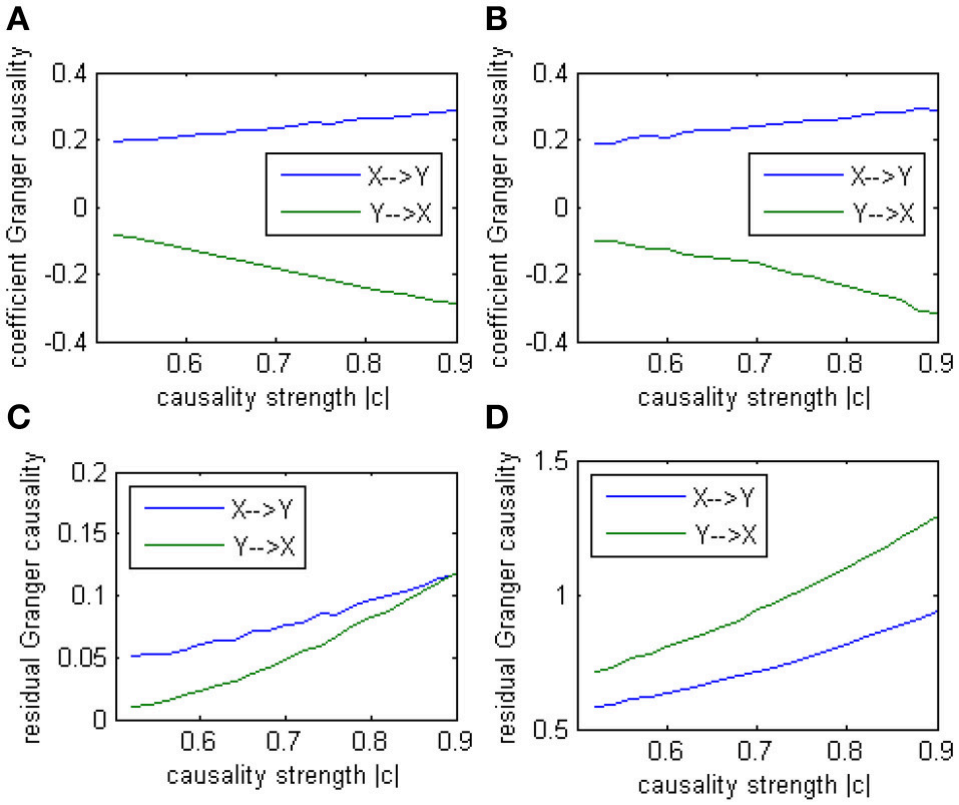
**Granger causality results from Model 1 and Model 1b by 100 simulations. (A)** result from Model 1b by signed path coefficient causality. **(B)** result from Model 1 by signed path coefficient causality. **(C)** result from Model 1b by traditional residual causality under optimal order. **(D)** result from Model 1 by traditional residual causality under optimal order. **(B,D)** are same as in Figure 5.

In this alternative model we had set two casual influence between X and Y on order-1: a positive influence as 0.186+0.005^*^k from *X* to *Y*, and a negative influence as −0.071−0.011^*^k from *Y* to *X* (1 ≤ *k* ≤ 20). And two autocorrelation as 0.5, whose actual value was not very important. After applying coefficient causality method to this model we could observe an almost identical causal relationship (Figure 7A) as in the Model 1 (Figure 7B), but by the residual Granger causality method, the real causal strength (computed under the optimal order) was far lower in Model 1b (Figure 7C) than in Model 1 (Figure 7D). This example demonstrated that we could not discriminate the two causal mechanisms from the results by the coefficient method, since under the order-1 autoregression a large amount of information on higher order had been neglected, that's why a similar relationship was extracted, even though the causal strengths in the two models were quite different and the coefficient signs were also opposite. For this reason we concluded the advisable method in this case was the traditional (no-signed) Granger causality measure, which was based on the residuals and did not indicate any excitatory/inhibitory relationship.

Model 2. A model with order-1 autocorrelation.

(8) $$\begin{array}{l} \left(\begin{array}{c} X_{t}\\ Y_{t} \end{array}\right)=\left(\begin{array}{cc} 0.18 & 0\\ 0 & 0.18 \end{array}\right)\left(\begin{array}{c} X_{t\;-\;1}\\ Y_{t\;-\;1} \end{array}\right)+\left(\begin{array}{cc} 0 & 0.8\\ -0.8 & 0 \end{array}\right)\left(\begin{array}{c} X_{t\;-\;2}\\ Y_{t\;-\;2} \end{array}\right)\\ \;+\left(\begin{array}{cc} 0 & c\\ 0 & 0 \end{array}\right)\left(\begin{array}{c} X_{t\;-\;3}\\ Y_{t\;-\;3} \end{array}\right)+\left(\begin{array}{c} \epsilon_{x,\;t}\\ \epsilon_{y,\;t} \end{array}\right) \end{array}$$

**Figure 8:**
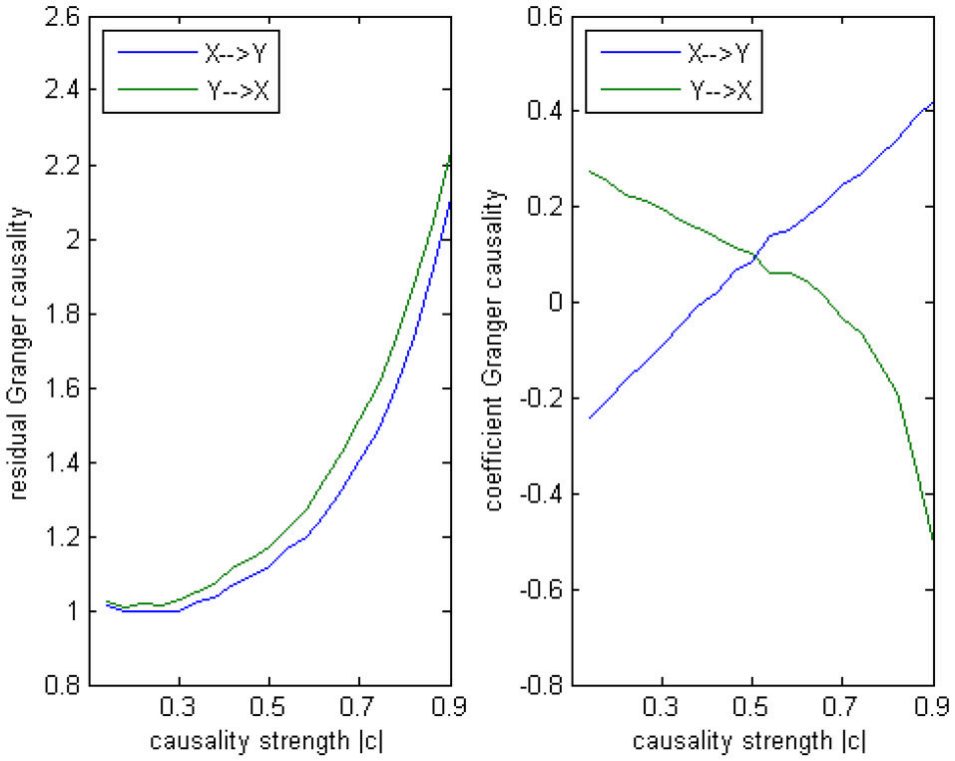
**Granger causality results from Model 2. Left:** result from traditional residual causality under optimal order. **Right:** result from order-1 signed path coefficient causality.

**Figure 9:**
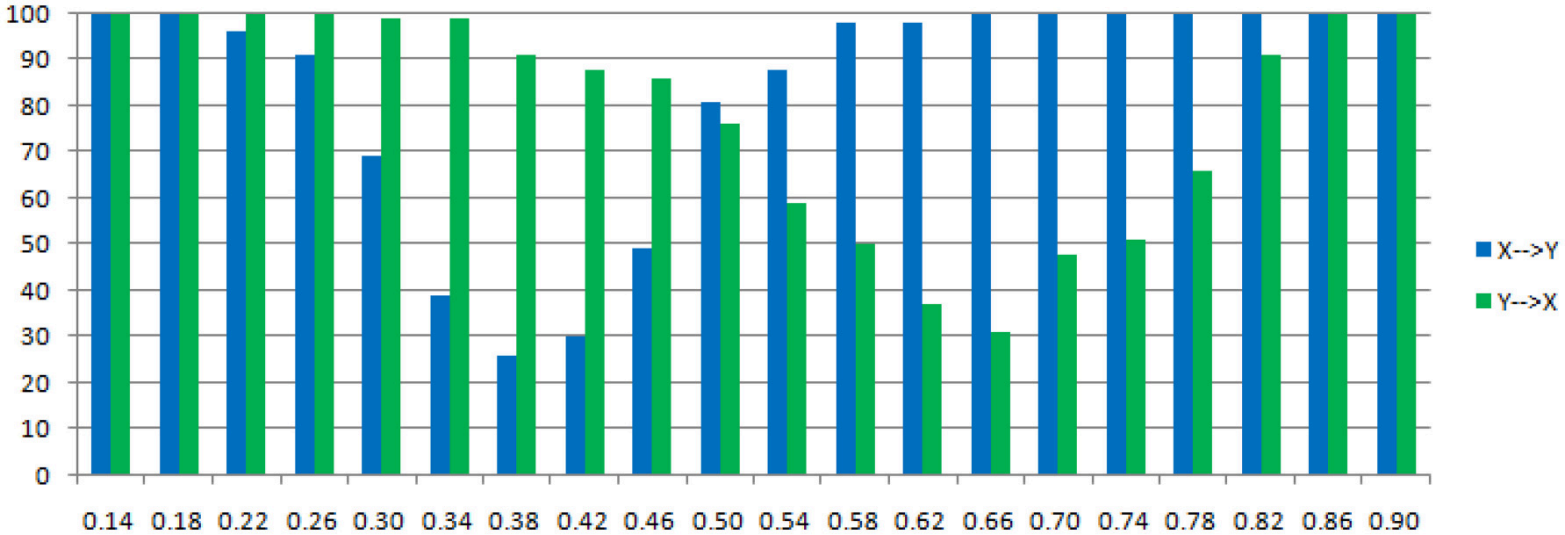
**Statistically significant percentage of connections between X and Y under permutation test for Model 2**. For each strength of c (x-axis), the number of significant results were showed by 100 simulations.

The main difference between Model 2 and Model 1 is the autocorrelation of *X* and *Y* on order-1 (with a strength 0.18), and the interactions on order-2 are a bit larger (+0.8/−0.8), c is still a negative influence on order-3 from *Y* to *X* whose strength varies from −0.1 to −0.9 with a step size of 0.04 (*c* = −0.1−0.04^*^k). Still we ran 100 trial simulations. An interest feature of this model was when the value of c was small, the coefficient causality method would give a pair of opposite signs compared with the result when the c was large (Figure 8, right part). As the strength of *c* increased, the causality from *X* to *Y* jumped from negative to positive, and the causality from *Y* to *X* changed from positive to negative, while the actual signs were all fixed. So the coefficient method failed to produce a consistent result. Even worse, when the influence *c* was of medium size, the value of one or both coefficients were relatively small so that they were not statistically significant, thus less likely to be detected (Figure 9), while on the other hand the causality derived from the residual method was steadily increasing with the strength of *c* (Figure 8, left part).

The total number of positive/negative coefficients computed by coefficient causality method in the 100 simulations was listed in Table 2. Comparing with the result in Model 1, there were always more positive coefficients than negative ones, the reason was due to the positive autocorrelation added in this model. And in actual fMRI time series this “positive autocorrelation” was a common condition, so this fact partly explained why the underfitted order-1 model leads to the positive bias of the coefficients in the fMRI result (Figure 1). From the results of Models 1 and 2 we could conclude that for some data generating process the signs of coefficients by an order-1 underfitted autoregression may change from positive to negative as other interactions on higher order varied, thus contradict the real underlying causal relationship. That explained why an excitatory/inhibitory assignment could hardly be operated, because given all the other coefficients fixed, just by changing the strength of an order-3 influence could radically alter the relationship from excitatory to inhibitory, therefore any attempt to define an interaction as excitatory/inhibitory would be impractical in this case. Moreover, since the information on higher orders were likely to be neglected by coefficient causality method, it tended to miss certain significant connections which could be identified by the residual causality method.

**Table 2:** **Granger causality results from Model 2**.

**C**	**#A+**	**#A−**	**AICc**	**BIC**
−0.52	100	100	3.28	2.34
−0.54	100	100	3.29	2.77
−0.56	100	100	3.23	2.98
−0.58	100	100	3.18	3
−0.60	104	96	3.19	3
−0.62	115	85	3.26	3
−0.64	137	63	3.32	3
−0.66	169	31	3.19	3
−0.68	183	17	3.2	3
−0.70	195	5	3.23	3
−0.72	185	15	3.18	3
−0.74	192	8	3.32	3
−0.76	174	26	3.32	3
−0.78	155	45	3.35	3
−0.80	141	59	3.14	3
−0.82	123	77	3.15	3
−0.84	112	88	3.24	3
−0.86	100	100	3.24	3
−0.88	100	100	3.22	3
−0.90	100	100	3.33	3

Number of positive/negative coefficients and the average AICc/BIC in 100 simulations under different causality strength c.

Model 3. An example that the coefficient method may give an approximately correct outcome.

(9) $$\begin{array}{l} \left(\begin{array}{c} X_{t}\\ Y_{t} \end{array}\right)=\left(\begin{array}{cc} 0 & 0.5\\ -0.5 & 0 \end{array}\right)\left(\begin{array}{c} X_{t\;-\;1}\\ Y_{t\;-\;1} \end{array}\right)+\left(\begin{array}{cc} 0 & 0\\ 0 & 0 \end{array}\right)\left(\begin{array}{c} X_{t\;-\;2}\\ Y_{t\;-\;2} \end{array}\right)\\ \;+\left(\begin{array}{cc} 0 & c\\ 0 & 0 \end{array}\right)\left(\begin{array}{c} X_{t\;-\;3}\\ Y_{t\;-\;3} \end{array}\right)+\left(\begin{array}{c} \epsilon_{x,\;t}\\ \epsilon_{y,\;t} \end{array}\right) \end{array}$$

**Figure 10:**
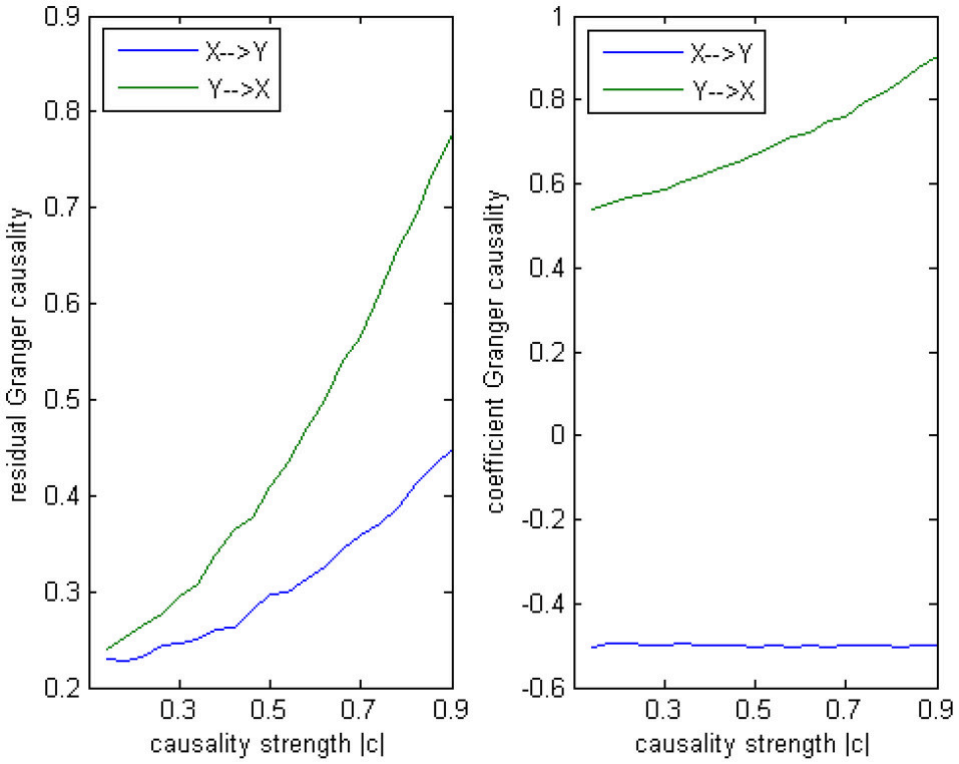
**Granger causality results from Model 3. Left:** traditional residual causality by optimal order. **Right:** signed path coefficient causality by order-1.

Model 3 is designed to give an example in which a correct conclusion could be derived by the coefficient method of an underfitted order-1 autoregression, to illustrate the signed path coefficient is applicable under certain conditions. In this model there is no order-2 interaction, but on order-1 two equal cross influences exist with the opposite signs (+0.5/−0.5), and on order-3 is a negative influence c from *Y* to *X* as same as in Model 2 (−0.1~−0.9). The AICc and BIC still provide an optimal order of 3. From the result by 100 simulations (Figure 10) we can observe the signs of coefficients are consistent with the real causal relationships, with an exact negative influence (−0.5) from *X* to *Y*, and an slightly increasing positive influence from *Y* to *X* (the trend is due to the varying c on order-3). In this example, the order-1 signed path coefficient Granger causality successfully captured the underlying causal structure despite the optimal order was 3. That's because although there existed an order-3 influence, its impact on the final results was not that critical compared with the dominated order-1 interactions between *X* and *Y*, hence this model did not greatly differ from an order-1 autoregressive model, for this reason the coefficient method could be fairly operationalized in this case.

## 5. Discussion and conclusion

In the present study we have focused primarily on the validity of a causality analysis technique: the signed path coefficient Granger causality, which has been applied by a number of fMRI researchers as an alternative method of Granger causality. In general, besides the formulations of original Granger causality and signed path coefficient causality which focus in time domain, there are other variants of causality measures derive from Granger causality that also rely on autoregressive modeling and are defined in frequency domain, among the most popular definitions are the spectral Granger causality (Geweke, 1982; Ding et al., 2006), direct transfer function (DTF) (Kaminski and Blinowska, 1991; Eichler, 2006), direct DTF (dDTF) (Korzeniewska et al., 2003), full frequency DTF (ffDTF) (Korzeniewska et al., 2003), partial directed coherence (PDC) (Sameshima and Baccalá, 1999; Baccalá and Sameshima, 2001), square partial directed coherence (sPDC) (Astolfi et al., 2006), and relative power contribution (RPC) (Yamashita et al., 2005). Although these methods perform diversely under different network situations, some previous studies have shown that Granger causality is robust enough to give an accurate prediction on neural network structure despite the complexity of network interconnections (Wu et al., 2011; Papana et al., 2013). Therefore, several neuroimaging studies have applied this approach to reveal the causality structure of their data.

However, can the signed path coefficient method be thought of as an efficient statistical Granger-like tool? Some researchers have used it as an equivalent form of traditional Granger causality for its ease of implementation and meaningful interpretation, but there are still some unclear aspects that need to be addressed. The most doubtful point is the signed path coefficient method to deduce causality relationship by an order-1 autoregression. Though the value of order is an important parameter for an autoregressive model, few authors mention it in their fMRI research, probably due to the usually long TR time of fMRI data. This fact has been noticed by some previous studies (Wu et al., 2013; Li et al., 2014) but without much discussion about the impact to the final analysis. Another reason why this issue was always neglected in the papers that adopted the signed path coefficient method is this method defining the property of a causal connection by the signs of coefficients, to distinguish whether an interaction is excitatory or inhibitory between brain regions, thus it is actually an intrinsic drawback. Since if we consider more higher orders to the autoregressive model, the definition of excitation/inhibition becomes invalid.

To investigate the impact of model order on the signed path coefficient method, we conducted a series of computations from resting-state fMRI data and simulation experiments. Firstly, we pointed out in fMRI data the optimal order of the autoregression model will not always be one, which actually depends on several other factors, e.g., the TR time and the number of ROIs to be analyzed. A shorter TR time or fewer time series of ROIs would often mean a higher optimal order, in this case the adoption of an universally order-1 was unreasonable. On the other hand, for the data with a relatively longer TR, the information criteria tend to give an optimal order of one, while the severe downsampling may corrupt the underlying causality (Seth et al., 2013), hence we meet the dilemma. Secondly, based on a few simulations that generated from process of a higher order, we demonstrated that under certain conditions the signed path coefficient method will present an outcome which contradict the real causal relationship of the data. Therefore, to some extent the definition of excitatory/inhibitory influence on the order-1 autoregression coefficient could only be constrained in the extreme narrow case of an actual order-1 data generation process, hence the application of the signed path coefficient method is not reliable to be generalized to any kind of fMRI analysis without limitation.

A constantly existing problem of the traditional (uniform) autoregression method in causality analysis is the number of model parameters increaseing rapidly (equal to the square of the total number of time series multiply by the model order) so the final model is prone to overparameterized which may lead to inefficient estimates, especially under the common sample size in fMRI. Therefore, it is difficult to uncover the real causal structure by this way (the “dimension curse”). And other sets of variables which provide information for the future state of target variable may worsen this situation (Stramaglia et al., 2012; Faes et al., 2014; Barrett, 2015). To improve the estimation to reach a well fitted model the parameters to be estimated should be properly reduced. Several shrinking strategies focus on this objective has been proposed by a number of researchers, in econometrics it is under the name “subset regression” (Penm and Terrell, 1984, 1986; Brüggemann and Lütkepohl, 2001; Brüggemann et al., 2003) which is based on sequential *t*-tests and model selection criteria or branch-and-bound strategy to cut subtrees (Gatu and Kontoghiorghes, 2005, 2006), and in physics it is called “Non-uniform Embedding” (Judd and Mees, 1998; Vlachos and Kugiumtzis, 2010; Kugiumtzis, 2013) which is based on the concept of transfer entropy (Schreiber, 2000), an information-theoretic analog to Granger causality, and the non-uniform state space reconstruction. Similar methods can also be found in climatology (Runge et al., 2012a,b; Hlinka et al., 2013; Radebach et al., 2013) and physiology (Faes et al., 2011, 2012), all of which are kind of variable specification and reduction procedures in which the most significant lag variables are picked up while others are restricted to zero in the model, thus reduce the dimensionality and shrink the state space. It has been shown all these sort of strategies can reserve the most relevant information in the data and give a parsimonious model (Wibral et al., 2014), thereby increase the forecast precision compare with the full autoregressive model. Nevertheless, by this strategy the higher order lags of variables are prone to be included in the analysis, and through our discussion above the signed path coefficient causality method can hardly be ameliorated, that means a critical part of indispensable higher order information will certainly be lost and the defect of signed path coefficient method can not be compromised.

Our current work concluded that the signed path coefficient method was not always suitable for fMRI data analysis compared to the traditional Granger causality method based on residuals, especially for the data with a shorter TR time and among fewer ROIs. We performed a series of simulations to indicate that the order-1 autoregressive model applied to data which derived by a higher order data generation progress may lead to incorrect results, thus any influence could not be properly defined as excitatory/inhibitory, which explained why this approach should be employed very carefully.

## Author contributions

In this work JZ has contributed the majority of analysis and synthesis. TJ and CL revised it critically and approved the version to be published.

## Funding

This work was partially supported by the National Key Basic Research and Development Program (973) (Grant No. 2011CB707801), the Strategic Priority Research Program of the Chinese Academy of Sciences (Grant No. XDB02030300), and the Natural Science Foundation of China (Grant No. 91132301) and the National Natural Science Foundation of China (Grant No. 11571308).

### Conflict of interest statement

The authors declare that the research was conducted in the absence of any commercial or financial relationships that could be construed as a potential conflict of interest.
